# Classification of Autism and Control Gait in Children Using Multisegment Foot Kinematic Features

**DOI:** 10.3390/bioengineering9100552

**Published:** 2022-10-14

**Authors:** Ashirbad Pradhan, Victoria Chester, Karansinh Padhiar

**Affiliations:** 1 Engineering Bionics Lab, Department of Systems Design Engineering, Faculty of Engineering, University of Waterloo, Waterloo, ON N2L6G2, Canada; 2 Andrew and Marjorie McCain Human Performance Lab, Faculty of Kinesiology, University of New Brunswick, Fredericton, NB E3B5A3, Canada

**Keywords:** autism, gait, multisegment foot, kinematics, machine learning

## Abstract

Previous research has demonstrated that children with autism walk with atypical ankle kinematics and kinetics. Although these studies have utilized single-segment foot (SSF) data, multisegment foot (MSF) kinematics can provide further information on foot mechanics. Machine learning (ML) tools allow the combination of MSF kinematic features for classifying autism gait patterns. In this study, multiple ML models are investigated, and the most contributing features are determined. This study involved 19 children with autism and 21 age-matched controls performing walking trials. A 34-marker system and a 12-camera motion capture system were used to compute SSF and MSF angles during walking. Features extracted from these foot angles and their combinations were used to develop support vector machine (SVM) models. Additional techniques-S Hapley Additive exPlanations (SHAP) and the Shapley Additive Global importancE (SAGE) are used for local and global importance of the black-box ML models. The results suggest that models based on combinations of MSF kinematic features classify autism patterns with an accuracy of 96.3%, which is higher than using SSF kinematic features (83.8%). The relative angle between the metatarsal and midfoot segments had the highest contribution to the classification of autism gait patterns. The study demonstrated that kinematic features from MSF angles, supported by ML models, can provide an accurate and interpretable classification of autism and control patterns in children.

## 1. Introduction

Autism spectrum disorder (ASD) is a complex neurodevelopmental disorder characterized by impaired social interactions and communication, and restricted and repetitive patterns of behaviors, interests, and activities (APA, 2013). ASD can also be characterized by various motor symptoms such as the delayed onset of motor milestones [1,2], hypotonia [3,4], and impaired postural control and balance [5]. These motor symptoms may affect a child’s ability to walk. Several studies have reported atypical gait patterns in children with autism [6,7], of which, several have highlighted atypical ankle range of motion [8], ankle kinematics [4,8,9,10,11,12], and ankle kinetics [8,9,10,13]. During gait, the foot and ankle function to attenuate shock at impact, provide stability and support to the lower limb, and aid in forward propulsion. Given the important role of the ankle/foot complex in gait, it is plausible that changes in foot function may result in impaired movement patterns. However, pediatric studies of gait patterns in ASD have modeled the foot as a single, rigid single foot segment (SSF) with one to two degrees of freedom. To date, no study has examined multisegment foot kinematic data in children with ASD during gait. Multisegment foot (MSF) mechanical models facilitate the investigation of the relative motion between foot subsegments (e.g., heel vs. forefoot), which can lead to a greater understanding of the role of the foot/ankle complex in ASD.

To further understand gait patterns in children with ASD versus neurotypical controls, there has been a recent increase in the number of studies using machine learning (ML) tools for classification based on combinations of kinematic and kinetic features [7,14,15,16,17,18,19]. Such studies facilitate the identification of discriminatory gait features in ASD versus neurotypical controls, which may lead to the development of automated gait classifiers, optimal treatment plans, improved functionality for children, and reduced health care costs. More research using ML tools is needed to further understand gait patterns and ankle/foot function in ASD.

### Overview of ML Studies for ASD Gait Assessment

Machine Learning (ML) has found numerous applications in the biomechanics field, such as pathological gait pattern detection [20] and fall detection in older adults [21]. Autism assessment is one such ML application that plays a role in the early detection of autism, as it can help clinicians shorten the diagnosis process and provide accurate results [22]. Some classification models have investigated social and communication impairments as the diagnostic criteria for ASD by utilizing brain image and clinical assessment data [23,24]. Other diagnosing characteristics such as the existence of motor deficits, specifically, atypical gait patterns could be explored further using classification models [7]. A previous study has suggested that disturbances due to walking can be used to diagnose ASD at an early age [25]. Gait patterns have been investigated using gait monitoring approaches such as sensor-based, marker-based and marker-less systems. The 3D marker-based system is considered the gold standard, from which kinematic features can be extracted to facilitate such data-driven approaches [7].

Previous research has investigated the classification of ASD and control gait patterns using temporal-spatial, kinematic, and kinetic features [7,14,15,16,17,18,19]. Ilias et al., (2006) observed that the fusion of all these features resulted in a classification accuracy of 95.80% (sensitivity 100%, specificity 85.00%) using a support vector machine (SVM) classifier [14]. Similarly, another study using only kinematic features from the hip, knee, and ankle joint, reported accuracy of 91.70% (sensitivity 93.30%, specificity 90.00%) using an artificial neural network (ANN) model [17]. In this study, a step-wise discriminant analysis feature selection was utilized for dimensionality reduction and demonstrated that the best performance could be attributed to four features, three of which were extracted from the ankle angle. Other dimensionality reduction techniques employed data transformations, such as principal component analysis and linear discriminant analysis [15] on three-dimensional joint position data, which resulted in an accuracy of 99.3% (sensitivity 99.66%, specificity 99.00%). More recently, a study extracted kinematic features from a markerless-based data acquisition [16] that resulted in a classification accuracy of 92% using a rough set classifier [19].

To date, all kinematic ML studies of gait patterns in autism have used SSF models. Given the number of gait studies that have highlighted atypical ankle mechanics [8,9,11,12,13,17], an investigation of the relative motion between foot segments via MSF kinematic models are needed. The combination of MSF kinematic data and ML tools will facilitate the exploration of more complex foot/ankle kinematic features for the classification of gait patterns in children with ASD. Contrary to the black-box nature of ML models, currently, interpretability tools are employed to analyze feature contribution and their ranking [26,27]. Hence, comparisons of MSF and SSF classification performance and feature rankings will provide insight into whether MSF models contribute more to the accurate classification of ASD than SSF models alone. This will provide researchers and clinicians with future directions of data collection and analysis protocols for autism gait assessment, taking into consideration factors such as sensory sensitivity in ASD and participant compliance.

The objectives of this study were to (1) investigate the classification of gait patterns in children with ASD versus neurotypical controls using MSF and SSF kinematic data, and (2) to determine the optimal MSF kinematic features for classifying gait patterns in children with ASD.

## 2. Materials and Methods

### 2.1. Participants

Nineteen children (*n* = 19) diagnosed with autism between the ages of 6–15 years participated in the study (16 males, 3 females; age = 10.47 ± 2.91 years; height: 1.42 ± 0.15 m; weight: 41.20 ± 17.00 kg) compared to a neurotypical control group consisting of twenty-one children (*n* = 21) between the ages of 6–16 years (11 males, 10 females, age: 11.38 ± 2.91 years; height: 1.49 ± 0.14 m; weight: 44.32 ± 16.36 kg). Participants were recruited through advertisements, emails, and word-of-mouth from the local community. Autism and control group characteristics are provided in Table 1. Parents voluntarily completed a questionnaire designed to identify possible injuries/diseases/disorders that could affect their child’s walking skills. No children were excluded from the study based on these responses. Further, no children in the autism group were classified as toe-walkers. Hypotonia was confirmed in 31.6% and the gross motor delay was confirmed in 26.3% of the children with autism.

*T*-tests were used to test for significant differences (*p* < 0.05) in height, weight, and age between the autism and control groups. No significant differences were found for height [F(1, 38) = 0.67, *p* = 0.17], weight [F(1, 38) = 0.85, *p* = 0.56], or age [F(1, 38) = 0.98, *p* = 0.34]. As no significant between-group differences were found, we consider the characteristics of the control group to approximate those of the autism group [28]. Parental consent and child assent were obtained prior to each child’s participation in the study. Ethical approval for this study was obtained from the University of New Brunswick Research Ethics Board.

### 2.2. Instrumentation

Data collection occurred at the Andrew and Marjorie McCain Human Performance Laboratory at the University of New Brunswick (UNB). A 12-camera Vicon T160 motion capture system (Oxford Metrics Group Ltd., Oxford, UK), sampling at 100 Hz, was used to track the three-dimensional trajectories of thirty-two retro-reflective spherical markers (9.5 mm diameter) placed directly on the skin of each participant. Six force plates (9281E, Kistler Instruments, Winterthur, Switzerland), embedded in the lab floor, were used to aid in the identification of key gait events (e.g., heelstrike, toe-off). The force plates were integrated and synchronized to the motion caption system through a 128-channel Vicon A/D board. Three-dimensional forces and moments were collected at a sampling frequency of 1000 Hz. Additionally, two high-speed Basler digital video cameras (Basler Inc., Ahrensburg, Germany) were used to obtain front and side images of each participant during gait trials. A weight scale and stadiometer were used to obtain anthropometric measures from each participant.

### 2.3. Experimental Protocol

**Figure 1:**
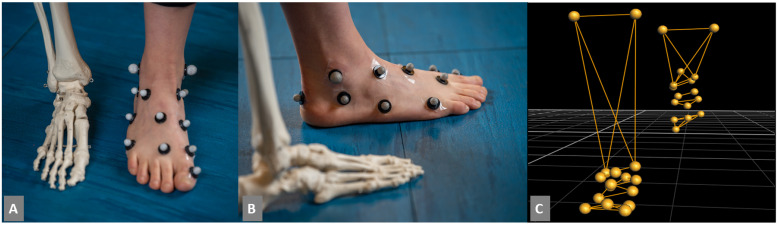
(**A**) and (**B**) Photograph of reflective marker locations on a participant’s feet, and (**C**) Single frame image of reconstructed three-dimensional marker trajectories from the Vicon motion capture system.

Thirty-two (*n* = 32) retro-reflective markers (Figure 1) were placed on the skin of each participant in accordance with a modified version of a previous multisegment foot model [29]. Modifications included: (1) the use of an additional cuboid marker (the cuboid was not assumed to coincide with the base of the fifth metatarsal, and (2) the formation of a neutral calcaneus using a laser-level technique to guide marker placement [30]. Several ‘warm-up’ trials were conducted to allow the participants to adjust to the markers and the lab environment. For each participant, a minimum of six successful dynamic trials (i.e., clean force plate strikes, marker visibility, accurate marker labels, and non-playful gait) for both the left and right leg were measured at their self-selected walking speed. Following the gait trials, participant weight and height were measured.

### 2.4. Biomechanical Data Processing

Three-dimensional trajectories of the reflective markers and force plate data were exported to Visual3D (v6, C-motion Inc., Boyds, MD, USA) for processing using a custom mechanical model. Marker trajectories were filtered with a 2nd order, low-pass Butterworth filter with a cut-off frequency of 8 Hz. For each participant, trial selection involved the computation of the mean walking speed across the 6 trials for each leg. The single gait cycle that most closely approximated the individual mean walking speed of all gait cycles (left or right) was selected as the single trial for analysis for each participant.

In accordance with a previous multisegment foot model [29], the rigid body model consisted of five segments: (1) the shank (tibia and fibula), (2) the total or single segment foot (SSF), (3) the calcaneus, (4) the midfoot, and (5) the forefoot (including all five metatarsal bones). The proximal phalanx of the hallux and the 1st, 2nd, and 5th metatarsal bones were modelled as line segments for the computation of planar angles. Joint angles were computed from the relative orientations of the embedded coordinate systems using Euler angles in an x-y-z sequence, corresponding to flexion/extension, adduction/abduction, and internal/external rotation. Joint angle data were normalized to 100% of the gait cycle. Planar angles included F2G: sagittal angle between the 1st metatarsal and the ground; S2G: sagittal angle between the 2nd metatarsal and the ground; S2F: transverse angle between 1st and 2nd metatarsals; V2G: sagittal angle of the 5th metatarsal and the ground; S2V: transverse angle between 5th and 2nd metatarsals; F2Ps: sagittal angle between 1st metatarsal bone and proximal phalanx; F2Pt: transverse angle between 1st metatarsal bone and proximal phalanx; and MLA: sagittal angle projection representing medial longitudinal arch.

The multisegment foot (MSF) features were extracted from the relative angles between 4 rigid segments, namely, the shank (Sha), calcaneus (Cal), midfoot (Mid), and forefoot (Met). The relative angle comparisons include Sha-Cal, Cal-Mid, Cal-Met, and Mid-Met. The SSF features were extracted from the relative angles between the rigid shank and total foot segment (Sha-Foot). For both the MSF and SSF mechanical models, the extracted features included the relative angle value at heel-strike (HS) and toe-off (TO), range-of-motion (ROM), maximum (MAX) and minimum (MIN) angles during stance (ST) and swing (SW) phase, and their corresponding time occurrences (TIME). For the feature extraction from planar angles, MAX and MIN values during stance were computed. A paired *t*-test was used to test for significant (*p* < 0.05) differences in features between left and right gait cycles across participants within each group. Results showed no significant differences (*p* > 0.05) or asymmetry between limbs for any features, therefore, all left and right cycles were pooled for each group. Independent *t*-tests for walking speed showed no significant differences (*p* > 0.05) between groups (autism: 1.25 ± 0.28 m/s; control: 1.21 ± 0.16 m/s).

Subsequently, throughout the document, the term feature(s) has been used to refer to the feature(s) extracted from the corresponding SSF/MSF/Planar angles. The MSF, SSF, and planar features, individually and combined, served as input to a support vector machine (SVM) classifier with a linear as well as a radial basis function (RBF) kernel. Further details are provided in Section 2.6.

### 2.5. Feature Selection

The recorded feature sets (as listed in the previous section) were used as input to feature selection techniques that were followed by retrospective classification of autism and control gait patterns. Feature selection methods were used to select a subset of the most contributing features from the original features set. This dimensionality reduction method was achieved by removing the redundant, irrelevant, or noisy features, and consequently, reducing the training time. Based on a preliminary analysis, we used a wrapper-based forward feature selection algorithm that follows an incremental greedy strategy for selecting the most contributing feature subset. Wrapper methods use a specific classifier with a cross-validation method to provide a classification score for each feature subset. The overall MSF + SSF feature set (#features = 233) and the planar feature set (#features = 40) was divided into sub-categories as explained in the following section and the forward feature section was employed separately for each category. The specific feature subset where the classification score plateaued was reported and further evaluation metrics were calculated.

### 2.6. Classification Models and Model Evaluation

A preliminary analysis investigated widely used machine learning models for biomechanical data, such as linear discriminant analysis, Naïve Bayes, k-nearest neighbors, and support vector machines. Based on the results, the support vector machine algorithm was chosen for the classification. For the SVM models, two different kernels: (1) linear and (2) radial basis function (RBF), were used to develop the models while the gamma and regularization parameters were varied [31]. Hyper-parameter tuning was performed to obtain the model development parameters for highest classification performance. All data analysis was performed using Python (PSF, Wilmington, DE, USA).

For the four sets of MSF relative angles (Sha-Cal, Cal-Mid, Cal-Met, Mid-Met), the classification models based on their respective features (e.g., max value at HS) were categorized into: (1) one-level and two-level combinations, and (2) three-level and four-level combinations, for easier presentation. Additionally, the all-combined feature set (MSF + SSF) was included in the second category. Similarly, for the eight planar angles, the classification models based on their respective features were categorized as (1) individual and (2) all combined. For each of the categories, sequential feature selection was performed, and each feature subset was modelled by the SVM classifiers. For each feature group, the subset with the highest classification performance was reported as shown below. For the evaluation of classification models according to their performance, leave-one-out cross-validation (LOOCV) was performed, and the averaged model evaluation metrics were reported. Some of the metrics investigated in this study are accuracy, specificity, sensitivity, positive predictive value (*PPV*), negative predictive value (*NPV*) [32], Matthew’s correlation coefficient (*MCC*) [33], and F1 score [34]. F1 score, which is the harmonic mean of precision and sensitivity is calculated as:

$$F1=\frac{2PPV\cdot sensitivity}{2PPV+sensitivity}=\frac{2TP}{2TP+FP+FN}$$

*MCC* score was calculated as:

$$MCC=\frac{TP=TN\cdot FP\cdot FN}{\sqrt{\left(TP+FP\right)\left(TP+FN\right)\left(TN+FP\right)\left(TN+FN\right)}}$$

where *TP* is true positive, *FP* is false positive, *TN* is true negative, *FN* is false negative. For example, if a model classifies all 40 participants (A = 19; C = 21) as control, the model’s accuracy is 52.5%, sensitivity is 0%, specificity is 100%, *PPV* is 0%, *NPV* is 58.5%, *F1* score is 0 and MCC is 0.

To compare the models in each category, a summed ranking (*SR*) approach was employed [21]. Each of the model evaluation metric was ranked from best to worst numerically as one to *N*, where *N* is the total number of models in each comparison category. Rankings from each of the metrics were summed and further ranked (best model corresponds to the lowest summed ranking). Therefore, three summed ranking analyses were performed: (1) SSF and MSF level-1 and level-2 feature combinations, (2) MSF level-3 and level-4 feature combinations, and (3) Planar feature combinations. Additionally, to summarize the findings, the averaged receiver operating characteristic (ROC) curve obtained from different combinations at each level was plotted. The area under the curve (*AUC*) was used to compare the multiple analyses, a final summed ranking was performed using the top-3 models from each of the above-mentioned combinations.

### 2.7. Model Interpretation

For interpretation of the ML models, explainable AI methods involving local and global importance were employed in the dataset. Local explainable methods, such as Shapley Additive exPlanations (SHAP) [27] were implemented to understand the feature’s importance. For each class label, the SHAP values for a particular input represent the importance of each feature in classifying the input sample to that class. Positive SHAP values indicate that the model tends to detect “autism” gait patterns, while negative SHAP values indicate that the model tends to detect “control” gait patterns. For global interpretability, the Shapley Additive Global importancE (SAGE) technique is implemented, which estimates feature importance by a global decomposition of the model loss across a whole data set [26]. The estimated SAGE value explains the influence of the features considering not only the model but also implicitly the data via the loss function. Therefore, while SHAP determines how much each feature contributes to individual prediction, SAGE determines the extent to which the model depends on each feature in the whole dataset. Both the SHAP and SAGE utilize an additive Shapley value which considers the correlation between variables, and hence can be further utilized to form feature groups.

Therefore, for our dataset, SSF feature group (#features = 47), MSF feature groups (#features = 186): Cal-Met, Cal-Mid, Mid-Met, and Sha-Cal, and the Planar feature groups (#features = 40): MLA, F2Ps, F2Pt, V2G, F2G, S2G, S2F, and S2V were formed for analyzing the feature importance. Subgroups comprising time variables and the 3D angle components were also investigated for feature importance.

## 3. Results

The Section 3 is organized into two parts: (A) classification models and model evaluation for reporting the classification performance of different SSF, MSF, and planar feature models, and (B) model interpretation results explaining the model predictions.

### 3.1. Classification Models and Model Evaluation

The evaluation metrics for the classification models based on level-1 and level-2 combinations of MSF features are provided in Table 2.

The classification models were ranked according to their SR scores. The models using SSF features (shown as Sha-Foot) in Table 2 were ranked 17th (accuracy = 0.838) and 18th (accuracy = 0.838). The top-ranked model among level 1–2 combinations was based on Cal-Met + Sha-Cal RBF features (accuracy = 0.938, F1 score = 0.943, specificity = 0.976). The highest sensitivity (0.947) was obtained by the second-ranked RBF model based on Mid-Met + Sha-Cal features. In the individual MSF feature group performance (level-1), it was observed that the highest accuracy was obtained by linear model based on Cal-Mid features (accuracy = 0.887, rank = 11) and the worst performing was model based on Sha-Cal features (accuracy = 0.762, rank = 26). Overall, the models based on level-2 combination (accuracy = 0.894 ± 0.038) performed significantly better (*p* < 0.001) than the level-1 combinations (accuracy = 0.821 ± 0.037). It was also observed that 8 of the top 10 models were using an RBF kernel SVM and the remaining two utilized a linear SVM. Overall, the RBF kernel models (accuracy = 0.880 ± 0.053) had higher accuracy than the linear models (accuracy = 0.851 ± 0.047). All the 26 models had an MCC greater than 0, which shows the classification of autism and control gait patterns were not random guesses.

Table 3 lists the evaluation metrics for the classification models based on level-3 and level-4 combinations of SSF and MSF features. The classification models were ranked based on their SR scores.

The top-ranked model was and RBF kernel SVM based on level-4 MSF parameters: Cal-Met, Cal-Mid, Mid-Met, and Sha-Cal (accuracy = 0.963, F1 score = 0.965, specificity = 0.976, sensitivity = 0.947). Similar results were obtained for the (SSF + MSF) RBF model, which is a combination of the single segment foot and multisegment foot features. The level-3 combination of Cal-Met + Mid-Met + Sha-Cal had similar accuracy but slightly lower specificity (0.952). Overall, the level-4 combination RBF model (accuracy = 0.963) did not perform significantly better as compared to the level-3 combination RBF (accuracy = 0.946 ± 0.017) models. Nine of the top ten models utilized the RBF kernel SVM. The RBF kernel models (accuracy = 0.950 ± 0.015) performed significantly better (*p* < 0.001) than linear SVM models (accuracy = 0.906 ± 0.025). All 18 models had an MCC greater than 0, which shows the classification of autism and control gait patterns were not random guesses.

Table 4 shows the evaluation metrics for the models based on planar features and their combinations. The best performing model based on individual planar feature groups (level-1) models was based on MLA features using an RBF kernel (accuracy = 0.762, F1 = 0.808). Combining all 8 planar feature groups improved the accuracy to 0.912. There was no significant difference (*p* = 0.229) between RBF kernel models (accuracy = 0.715 ± 0.039) and linear models (accuracy = 0.693 ± 0.029).

**Figure 2:**
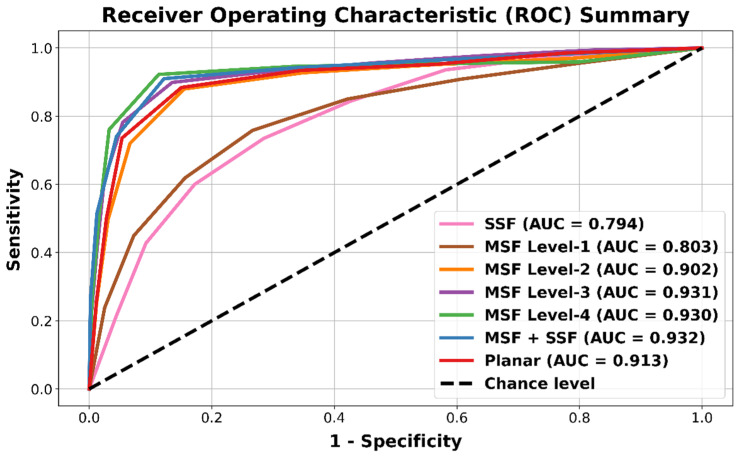
ROC curve summary plot for comparing different SSF, MSF feature groups combination levels (level 1–4), combined MSF + SSF features, and planar features. The MSF level-1, level-2, and level-3 curves are obtained by averaging the performance of different MSF feature combinations in each category. The SSF, MSF level-4, MSF + SSF combined and planar features ROC curves are obtained from specific feature-set based models. The area under the curve (AUC) values are provided in the legend entry.

The ROC summary plots were used to compare different combinations of SSF, MSF, and planar feature groups as shown in Figure 2. It was observed that the SSF features performed the worst (AUC = 0.794). The averaged MSF level-1 feature groups had a similar lower performance (AUC = 0.803), however, the performance increased for MSF level-2 (AUC = 0.902) and further for MSF level-3 (AUC = 0.931). The performance plateaued as MSF level-4 did not contribute to a better classification (AUC = 0.930). Similar results were obtained for combining the MSF features with the SSF features (AUC = 0.932), where the performance was similar to MSF level-3. The planar features resulted in lower performance (AUC = 0.913) as compared to the higher-level MSF combinations.

### 3.2. Model Interpretation

**Figure 3:**
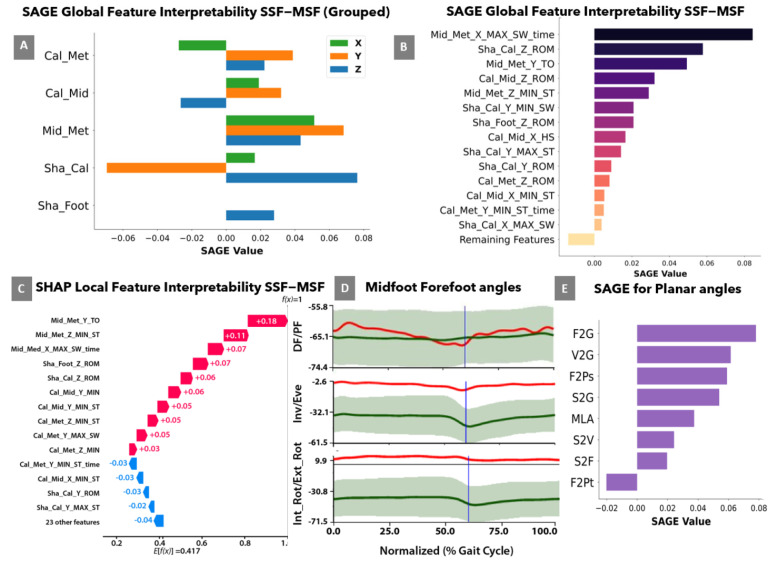
(**A**) SAGE values for feature groups for dorsiflexion/plantarflexion, inversion/eversion, and internal/external rotation angles corresponding to the X, Y and Z components, respectively of MSF and SSF features, (**B**) SAGE values for individual feature ranking showing the 15 top most ranked features (**C**), SHAP waterfall plot for local interpretation of a sample autism case (A14), red arrow shows prediction contribution towards the autism category and blue arrows shows prediction contribution towards the control category (**D**) Mid-Met angle values in the three planes for A14 (shown by red line vs. control mean +/− 1SD shown by green), and (**E**) SAGE values for planar feature groups.

For global interpretation, Figure 3A,B shows the SAGE feature groups and individual feature ranking, respectively. It was observed that max/min and time to max/min biomechanical features for Mid-Met contributed the most to the model (SAGE > 0.04). From the individual feature ranking, it was observed that the features associated with the Mid-Met segment ranked three out of five. Sha-Foot features did not appear until feature-rank 7 (i.e., MSF features are #1–6 and contribute the most to the model compared to SSF). For local interpretation, a SHAP water fall plot (Figure 3C) was provided to briefly display feature contribution for an individual autism case (A14). The direction and magnitude of a feature are presented as follows: the right arrows indicate the contribution of a feature towards the autism category (red) and the left indicates a contribution toward the control category (blue), and the length of the arrows determines the magnitude of a feature effect. The results show that the top-3 features are from the Mid-Met segment (SHAP values > 0.06). This is similar to the global interpretability obtained using SAGE value. Thirteen of the top-14 features are MSF parameters, with only one SSF parameter (rank = 4).

Figure 3D shows the individual MSF kinematic results of participant A14 (shown by the red line) for the Mid-Met segment. The dorsiflexion/plantarflexion, inversion/eversion, and internal/external rotation angles (top-bottom) for the Mid-Met segment are shown in the respective order. The Mid-Met angle shows decreased eversion and increased internal rotation across the entire cycle compared to control data. This result corroborates the findings using SAGE and SHAP plots. Figure 3E illustrates the ranking of planar feature groups using the SAGE grouping values. Results indicate that the F2G feature group had the highest SAGE value (0.08) as compared to other feature groups.

## 4. Discussion

### 4.1. Classification Evaluation of Autism Gait Patterns

To date, no previous studies have examined the classification of MSF versus SSF kinematics in children with autism versus neurotypical controls. Table 2 presented the rankings of SSF and MSF group level 1–2 combinations. The SSF models had an accuracy of 0.838 and ranked low compared to the other MSF features and their combinations. Additionally, all of the top ten models had level-2 combinations (0.90 < accuracy < 0.938), i.e., the feature set comprised of two MSF feature groups. Specifically, the RBF for level-2 combinations of MSF features (accuracy = 0.918 ± 0.015) was greater than level-1 combinations of MSF features (accuracy = 0.820 ± 0.027). This suggests that a combination of two MSF features might provide additional variation to the data and can result in higher accuracy than the SSF model for classifying gait patterns in children with autism.

This trend was also observed while analyzing MSF level-3 and level-4 combinations (Table 3). It was observed that out of the level-3 and level-4 combinations of MSF features, maximum accuracy of 0.963 could be achieved for classifying autism gait patterns. Specifically, three models: level-4 MSF features, MSF + SSF (level-5), and Cal-Mid + Mid-Met + Sha-Cal (level-3), had the highest accuracy of 0.963 among all models. This was greater than other level-3 combinations of MSF with an average accuracy of 0.946 ± 0.017 using an RBF kernel. This suggests that although a level-4 combination of all MSF features, as well as a further combination with SSF, features, resulted in the highest accuracy of 0.963, comparable classification performance can be achieved using a combination of three MSF feature groups only. This performance was greater than any of the level-1 and level-2 MSF feature combinations. The high MCC value of all Table 3, models (MCC > 0.749), suggests that the classification results were reliable and balanced between both the classes.

The combined planar features model (Planar_All), which used features from all 8 planar groups, had the highest accuracy (0.912) using an RBF kernel. The linear model counterpart resulted in a slightly lower accuracy (0.875). The MLA RBF model resulted in an accuracy of 0.762, sensitivity of 0.553, and specificity of 0.952, which although the highest among all the individual planar feature models, was lower than the combined models. This suggests that a combination of planar feature groups can present a higher accuracy for classifying autism gait patterns.

Table 5 provides a summary of all the top performing models, which also corroborates the ROC summary plot provided in Figure 2.

From both Table 5 and Figure 2, it can be confirmed that the MSF combination of features resulted in higher accuracy than SSF features for classifying autism gait patterns. Specifically, the level-3 combination models had very high average classification performance (accuracy = 0.946, AUC = 0.931).

While comparing the RBF SVM and linear SVM models, it was observed that for level-3 and level-4 combinations, the RBF kernel models (accuracy = 0.950 ± 0.015) performed significantly higher (*p* < 0.001) than linear SVM models (accuracy = 0.906 ± 0.025). It was also observed that for the level-2 combinations, the RBF kernel (accuracy = 0.918 ± 0.015) performed significantly higher (*p* < 0.001) than the linear SVM (0.870 ± 0.039). For level-1 combinations, the accuracy of the RBF kernel (0.820 ± 0.027) was not significantly different (*p* = 0.678) than the linear SVM (0.822 ± 0.048). This suggests that with increasing feature space, the data tends to be linearly inseparable and thus a more complex non-linear hyperplane might be better suited for classification.

### 4.2. Feature Rankings and Model Interpretability

From Table 2, Table 3, Table 4 and Table 5, a particular trend for the Mid-Met features was observed. The level-1 feature combinations (Mid-Met only) resulted in lower accuracy (0.775 for RBF, ranked 24 in Table 2) compared to the other three MSF feature groups (such as Cal-Mid, accuracy = 0.887 for linear, ranked 11 in Table 2). However, it was observed that a level-2 combination of Mid-Met and Sha-Cal resulted in a higher accuracy (0.938 for RBF, ranked 2 in Table 2) and a level-3 combination of Cal-Mid, Mid-Met, and Sha-Cal resulted in even higher accuracy (0.963 for RBF, highest accuracy in Table 3). This suggests that although the Mid-Met features have lower classification performance independently, they provide relatively higher classification performance with a combination of other MSF features.

The model interpretability analysis using SHAP and SAGE is discussed next which suggests that the Mid-Met features contributed most to the best-performing models (Figure 3A–D). It was observed that max/min and time to max/min biomechanical features for Mid-Met contributed the most to the model (SAGE > 0.04). The features related to the Mid-Met segment were the magnitude of inversion/eversion at toe-off (Mid_Met_AngleY_TO) as feature #1 and the minimum internal/external rotation during stance (Mid_Met_AngleZ_Min_ST) as feature #2. In this individual case, the forefoot showed decreased eversion and internal rotation with respect to the midfoot across the entire cycle compared to control data (Figure 3B,C). As shown in Figure 3, the primary feature contributing to the classification of this participant as having an autism gait pattern is the magnitude of eversion of the midfoot-forefoot at toe-off (Mid_Met_AngleY_TO), followed by the magnitude of external rotation (or minimum internal rotation) during stance (Mid_Met_AngleZ_MIN_ST). These findings suggest differences in the kinematic coupling between foot segments for autism gait patterns versus control that can impact foot stability and propulsion.

For the planar angle comparisons, Figure 3E suggests that F2G contributed most to the best-performing planar features model (accuracy = 0.912, AUC = 0.913). As such, the upward/downward orientation of the first metatarsal relative to the ground during stance contributes to the differentiation of ASD versus neurotypical gait, with ASD showing significantly (*p* < 0.05) reduced mean max values in stance (ASD: 84.15 ± 15.29; Control: 91.38 ± 10.47). While these planar projection angles are less reliable, they affirm the need to examine forefoot dynamics.

### 4.3. Comparison with Previous Studies

To our knowledge, this is the first paper to analyze multisegment foot kinematics in children with ASD. To date, it is also the first to apply machine learning techniques to multisegment foot kinematic data for any population. As such, comparison to previous work is limited. A previous study [17], examining autism gait classification using hip, knee, and ankle (SSF) kinematics using an ANN model, reported an accuracy of 91.7%. The present study reported lower accuracy for SSF features (83.8%), however, this may be due to the use of only ankle kinematic features and an RBF SVM model for classification. Moreover, MSF feature combinations were able to achieve a higher accuracy of 96.3%. Three ankle features from the previous study [17], namely, MAX ankle plantarflexion during stance, and MAX internal and external foot rotation across the gait cycle were included in the present study. Individual feature rankings (Figure 3B) showed that the internal/external rotation range of motion for the SSF model did not appear until feature-rank 7. Therefore, MSF features provide insight into complex foot mechanics in ASD, while also providing high classification accuracies compared to most SSF models.

Hasan et al. [35] also compared the performance of multi-joint kinematic versus kinetic features using two different classifiers. Linear discriminant analysis (LDA) and quadratic discriminant analysis (QDA) were used to classify ASD gait patterns from a statistically reduced set of nine kinematic and sixteen kinetic features, which included SSF parameters, namely MAX ankle dorsiflexion angle in stance, MAX plantarflexion angle in swing, MAX sagittal and transverse moments, and MAX eccentric ankle power. Results suggested that the LDA classifier with kinetic gait features was the most effective for classifying ASD gait patterns (82.5%). This differed from previous work [17], which achieved higher classification rates using ankle and knee kinematic features only. Similarly, the highest reported classification accuracy of 99.3% was achieved using principal component analysis and linear discriminant analysis for data reduction on full-body three-dimensional joint position data in children with ASD and controls [15]. A recent study extracted kinematic features from a markerless-based data acquisition [16] that resulted in a classification accuracy of 92% using a rough set classifier [19]. In contrast, previous research has investigated the classification of ASD and control gait patterns using temporal-spatial, kinematic, and kinetic features [7,14,15,16,17,18]. High classification accuracy of 95.8% has been achieved by combining all of these features using a support vector machine (SVM) classifier [14]. Variability in feature sets, data reduction and classification techniques, and accuracy suggest there is more research needed to reach a consensus on which gait parameters best discriminate ASD gait from neurotypical controls. Interestingly, this research also shows that gait patterns in ASD and controls can be successfully grouped.

## 5. Conclusions

Gait patterns in children with autism were compared to controls using linear and RBF SVM models based on planar, SSF, and MSF kinematic features. Results suggested that combinations of MSF kinematic features retrospectively classified autism patterns with an accuracy of 96.3%, which is higher than using SSF kinematic features alone (83.8%). The RBF kernel models had an overall better performance than the linear models. Additionally, through interpretability approaches, we identified that features of the relative angles between the metatarsal and midfoot had the highest contribution to the classification of autism gait patterns. Furthermore, the performance plateaued for a combination of three MSF features. The combination of all planar features resulted in an accuracy of 91.2%, with the F2G contributing the highest. This study demonstrated that kinematic features from a multi-segment foot model, supported by ML models, can provide an accurate classification of gait patterns in children with autism versus controls. Further research is needed using higher sample sizes to validate the ML model. Additional kinematic and kinetic features, as well as EMG data, should also be considered for future work. Additional markerless motion capture studies may also benefit children with sensory sensitivities and yield more natural movement patterns. The application of ML models, combined with model interpretability, can facilitate an increased understanding of gait patterns in autism, and in turn, the potential for optimized treatment programs and improved function and quality of life.

## Figures and Tables

**Table 1:** Participant Demographics (Mean ± Std. Dev.).

Characteristics	Autism (*n* = 19)	Control (*n* = 21)
Age (years)	10.47 ± 2.91	11.38 ± 2.91
Height (m)	1.42 ± 0.15	1.49 ± 0.14
Weight (kg)	41.20 ± 17.00	44.33 ± 16.36
Gender (#M, #F)	(16 M; 3 F)	(11 M; 10 F)

**Table 2:** Classification models based on level-1 and level-2 combinations of SSF and MSF features.

Segment	Method	Accuracy	Sensitivity	Specificity	*PPV*	*NPV*	*F1*	*MCC*	*SR*
Cal-Met + Sha-Cal	RBF	0.938	0.895	0.976	0.971	0.911	0.943	0.877	15
Mid-Met + Sha-Cal	RBF	0.938	0.947	0.929	0.923	0.951	0.94	0.875	23
Cal-Met + Sha-Foot	Linear	0.925	0.868	0.976	0.971	0.891	0.932	0.853	27
Cal-Mid + Mid-Met	RBF	0.925	0.895	0.952	0.944	0.909	0.930	0.850	34
Cal-Met + Cal-Mid	Linear	0.925	0.842	1	1	0.875	0.933	0.858	34
Sha-Cal + Sha-Foot	RBF	0.925	0.921	0.929	0.921	0.929	0.929	0.850	35
Cal-Met + Mid-Met	RBF	0.912	0.868	0.952	0.943	0.889	0.920	0.826	47
Cal-Met + Cal-Mid	RBF	0.912	0.921	0.905	0.897	0.927	0.916	0.825	54
Cal-Met + Sha-Foot	RBF	0.900	0.868	0.929	0.917	0.886	0.907	0.800	61
Cal-Mid + Sha-Cal	RBF	0.900	0.816	0.976	0.969	0.854	0.911	0.807	63
Cal-Mid	Linear	0.887	0.868	0.905	0.892	0.884	0.894	0.774	75
Cal-Met + Sha-Cal	Linear	0.887	0.868	0.905	0.892	0.884	0.894	0.774	75
Mid-Met + Sha-Cal	Linear	0.875	0.842	0.905	0.889	0.864	0.884	0.750	93
Cal-Mid + Mid-Met	Linear	0.850	0.763	0.929	0.906	0.812	0.867	0.705	104
Cal-Met	Linear	0.838	0.737	0.929	0.903	0.796	0.857	0.682	113
Sha-Cal	RBF	0.838	0.868	0.810	0.805	0.872	0.840	0.677	120
Sha-Foot	Linear	0.838	0.842	0.833	0.821	0.854	0.843	0.675	120
Sha-Foot	RBF	0.838	0.842	0.833	0.821	0.854	0.843	0.675	120
Cal-Mid	RBF	0.838	0.763	0.905	0.879	0.809	0.854	0.678	121
Cal-Met	RBF	0.812	0.632	0.976	0.960	0.745	0.845	0.655	122
Sha-Cal + Sha-Foot	Linear	0.838	0.816	0.857	0.838	0.837	0.847	0.674	125
Cal-Met + Mid-Met	Linear	0.838	0.789	0.881	0.857	0.822	0.851	0.675	125
Cal-Mid + Sha-Cal	Linear	0.825	0.816	0.833	0.816	0.833	0.833	0.649	145
Mid-Met	RBF	0.775	0.816	0.738	0.738	0.816	0.775	0.554	164
Mid-Met	Linear	0.787	0.789	0.786	0.769	0.805	0.795	0.575	165
Sha-Cal	Linear	0.762	0.658	0.857	0.806	0.735	0.791	0.528	170

Linear: Linear SVM, RBF: Radial Basis Function kernel SVM; Sha: Shank, Cal: Calcaneus, Mid: Midfoot, Met: Metatarsus, Foot: Total Foot; *NPV*: Negative Predictive Value: *PPV*: Positive Predictive Value, MCC: Matthew’s Correlation Coefficient, SR: Summed Rank.

**Table 3:** Classification models based on level-3, 4 and 5 combinations of SSF and MSF features.

Segment	Method	Accuracy	Sensitivity	Specificity	*PPV*	*NPV*	*F1*	*MCC*	*SR*
MSF (combined)	RBF	0.963	0.947	0.976	0.973	0.953	0.965	0.925	15
MSF + SSF	RBF	0.963	0.947	0.976	0.973	0.953	0.965	0.925	15
Cal-Mid + Mid-Met + Sha-Cal	RBF	0.963	0.974	0.952	0.949	0.976	0.964	0.925	21
Cal-Mid + Sha-Cal + Sha-Foot	RBF	0.963	0.974	0.952	0.949	0.976	0.964	0.925	21
Cal-Met + Cal-Mid + Sha-Cal	RBF	0.950	0.895	1	1	0.913	0.955	0.904	35
Cal-Met + Sha-Cal + Sha-Foot	RBF	0.950	0.895	1	1	0.913	0.955	0.904	35
Cal-Met + Cal-Mid + Mid-Met	RBF	0.950	0.921	0.976	0.972	0.932	0.953	0.901	39
MSF + SSF	Linear	0.950	0.921	0.976	0.972	0.932	0.953	0.901	39
Cal-Met + Cal-Mid + Mid-Met	Linear	0.938	0.921	0.952	0.946	0.930	0.941	0.875	57
Cal-Met + Mid-Met + Sha-Cal	RBF	0.938	0.947	0.929	0.923	0.951	0.940	0.875	58
Cal-Met + Cal-Mid + Sha-Foot	RBF	0.912	0.895	0.929	0.919	0.907	0.918	0.825	76
Cal-Mid + Sha-Cal + Sha-Foot	Linear	0.912	0.895	0.929	0.919	0.907	0.918	0.825	76
MSF (combined)	Linear	0.912	0.895	0.929	0.919	0.907	0.918	0.825	76
Cal-Met + Cal-Mid + Sha-Foot	Linear	0.900	0.842	0.952	0.941	0.870	0.909	0.803	93
Cal-Met + Mid-Met + Sha-Cal	Linear	0.887	0.895	0.881	0.872	0.902	0.892	0.775	103
Cal-Met + Sha-Cal + Sha-Foot	Linear	0.887	0.868	0.905	0.892	0.884	0.894	0.774	108
Cal-Mid + Mid-Met + Sha-Cal	Linear	0.875	0.816	0.929	0.912	0.848	0.886	0.752	113
Cal-Met + Cal-Mid + Sha-Cal	Linear	0.875	0.868	0.881	0.868	0.881	0.881	0.749	119

**Table 4:** Classification models based on planar features.

Segment	Method	Accuracy	Sensitivity	Specificity	*PPV*	*NPV*	*F1*	*MCC*	*SR*
Planar_All	RBF	0.912	0.868	0.952	0.943	0.889	0.920	0.826	7
Planar_All	Linear	0.875	0.868	0.881	0.868	0.881	0.881	0.749	21
MLA	RBF	0.762	0.553	0.952	0.913	0.702	0.808	0.557	30
S2G	RBF	0.762	0.605	0.905	0.852	0.717	0.800	0.539	33
V2G	RBF	0.738	0.579	0.881	0.815	0.698	0.779	0.486	46
MLA	Linear	0.738	0.526	0.929	0.870	0.684	0.788	0.502	48
F2G	RBF	0.725	0.553	0.881	0.808	0.685	0.771	0.462	58
S2V	Linear	0.725	0.579	0.857	0.786	0.692	0.766	0.457	62
S2G	Linear	0.700	0.421	0.952	0.889	0.645	0.769	0.447	64
S2V	RBF	0.713	0.658	0.762	0.714	0.711	0.736	0.423	70
F2Ps	Linear	0.700	0.711	0.690	0.675	0.725	0.707	0.401	74
F2G	Linear	0.700	0.500	0.881	0.792	0.661	0.755	0.415	76
F2Ps	RBF	0.700	0.579	0.810	0.733	0.680	0.739	0.401	80
S2F	Linear	0.662	0.395	0.905	0.789	0.623	0.738	0.351	91
F2Pt	Linear	0.662	0.684	0.643	0.634	0.692	0.667	0.327	96
V2G	Linear	0.662	0.447	0.857	0.739	0.632	0.727	0.336	99
F2Pt	RBF	0.662	0.658	0.667	0.641	0.683	0.675	0.324	99
S2F	RBF	0.662	0.579	0.738	0.667	0.660	0.697	0.322	102

**Table 5:** Summary of top-performing models.

Segment	Method	Accuracy	Sensitivity	Specificity	*PPV*	*NPV*	*F1*	*MCC*
Cal-Met + Cal-Mid + Mid-Met + Sha-Cal	RBF	0.963	0.947	0.976	0.973	0.953	0.965	0.925
All (MSF + SSF)	RBF	0.963	0.947	0.976	0.973	0.953	0.965	0.925
Cal-Mid + Mid-Met + Sha-Cal	RBF	0.963	0.974	0.952	0.949	0.976	0.964	0.925
Cal-Met + Sha-Cal	RBF	0.938	0.895	0.976	0.971	0.911	0.943	0.877
Mid-Met + Sha-Cal	RBF	0.938	0.947	0.929	0.923	0.951	0.94	0.875
Cal-Met + Sha-Foot	Linear	0.925	0.868	0.976	0.971	0.891	0.932	0.853
Planar-All	RBF	0.912	0.868	0.952	0.943	0.889	0.92	0.826
Planar-All	Linear	0.875	0.868	0.881	0.868	0.881	0.881	0.749
Sha-Foot	RBF	0.838	0.842	0.833	0.821	0.854	0.843	0.675

## Data Availability

Data sharing not applicable.
